# Absolute and relative reliability of *SCRuM* test battery components assembled for schoolboy rugby players playing competitive rugby in low-resource settings: A pragmatic in-season test-retest approach

**DOI:** 10.17159/2078-516X/2021/v33i1a12220

**Published:** 2021-02-15

**Authors:** M Chiwaridzo, C Tadyanemhandu, NS Mkumbuzi, JM Dambi, GD Ferguson, BC Smits-Engelsman

**Affiliations:** 1 University of Zimbabwe, Faculty of Medicine and Health Sciences, Department of Primary Health Care Sciences, Rehabilitation Unit, Harare, Zimbabwe; 2 University of Cape Town, Faculty of Health Sciences, Department of Health and Rehabilitation Sciences, Division of Physiotherapy, Cape Town, South Africa; 3 Midlands State University, Faculty of Medicine and Health Sciences, Physiotherapy Department, Gweru, Zimbabwe; 4 University of Cape Town, Health Through Physical Activity, Lifestyle and Sports Research Centre, Department of Human Biology, Cape Town, South Africa

**Keywords:** intraclass correlation, reproducibility, rugby union, Under 19, Zimbabwe, Africa

## Abstract

**Background:**

Schoolboy rugby is a popular sport which forms the bedrock of rugby development in many African countries, including Zimbabwe. With burgeoning talent identification programmes, the development of multi-dimensional, logically-validated, and reliable test batteries is essential to inform the objective selection of potentially talented young rugby athletes.

**Objectives:**

This study sought evidence on the absolute and relative test-retest reliability of the component test items in the newly-assembled *SCRuM* test battery.

**Methods:**

Utilising a pragmatic test-retest experimental design, a sample of 41 Under-19 schoolboy players playing competitive rugby in the elite Super Eight Schools Rugby League in Harare, Zimbabwe, participated in the study.

**Results:**

Physiological and game-specific skills tests which showed good to excellent relative reliability and acceptable absolute reliability, included: 20 m and 40 m speed, L-run, Vertical Jump (VJ), 60 s Push-Up, 2 kg Medicine Ball Chest Throw test (2 kg MBCT), Wall Sit Leg Strength test (WSLS), Repeated High Intensity Exercise test (RHIE), One Repetition Maximum Back Squat (1-RM BS) and Bench Press tests (1-RM BP), Yo-Yo Intermittent Recovery Level 1 test (Yo-Yo IRT L1), Tackling Proficiency test, Passing Ability Skill test and Running and Catching Ability skill test.

**Conclusion:**

All these tests are reliable and warrant inclusion in the* SCRuM* test battery for possible profiling of U19 schoolboy rugby players during the ‘in-season’ phase provided there is adequate participant familiarisation and test standardisation. The test-retest ICCs and measurement errors are generalisable to other young athletes in this population, making the tests useful for the evaluation of training and developmental effects of the measured constructs.

Increased competition demands worldwide at the elite senior level have prompted professional rugby union (RU) clubs and national RU governing bodies to invest in talent identification (TID) and long-term junior development programmes.^[1–3]^ These efforts have produced a pool of young rugby players with the potential to become successful future elite athletes, strengthening the growth and development of rugby. The process of TID is dependent on screening tests that measure the important characteristics of rugby players. The tests should be practically feasible and have acceptable psychometric properties. However, there are many test batteries available in the literature profiling young rugby players with heterogeneous compositions ^[4]^ and unclear details on the measurement properties of the constituent tests.

Regardless of age and playing standards, RU is a physically and technically challenging sport requiring commensurate physiological adaptations and specialised training of rugby-specific skills for optimal performances. ^[1]^ A combination of appropriate anthropometric qualities, physiological characteristics, and rugby-specific skills defines the key attributes warranted by participants for effective performances. Test batteries that are logically validated to the needs of the young rugby players, which also contain practically feasible and reliable tests are more likely to be relevant for use in the TID programs. In addition, coaches, strength and conditioning experts and sports scientists can use them for longitudinal monitoring of athletic motor skills, technical performances, and responses to injury rehabilitation. Cross-sectionally, such test batteries can provide data on players’ competency levels assisting in player team selection, and an individual athlete’s profile in terms of anthropometric, physiological and game-specific characteristics. Therefore, following the development of the first version of the *SCRuM* (School Clinical Rugby Measure) test battery and subsequent evaluation of face recognition methods, logical validity and practical feasibility of the component test items ^[4–6]^, the specific objectives of this study were to identify test items in the *SCRuM* test battery with an acceptable coefficient of variation and high intraclass correlation coefficient (ICC) as a measure of absolute and relative reliability among a sample of young rugby players.

## Methods

### Study design, research setting and participants

Experimentally, the study was conducted as a test-retest reliability study using Under 19 (U19) schoolboys playing competitive rugby in Harare, Zimbabwe. The only pathway for junior RU development in Zimbabwe is within the school system. In a bid to promote and strengthen junior rugby in high schools, the Ministry of Primary and Secondary Education in Zimbabwe, established the Super Eight Schools Rugby League (SESRL). The SESRL features the eight most competitive rugby-playing government (n=2) and private (n=6) high schools across the country and is generally considered as the “elite” rugby-playing league. All the SESRL schools have a local reputation for a strong and long-standing culture of playing competitive rugby. ^[4]^ Annually, the SESRL produces U19 rugby players capable of joining adult professional clubs.

Using tables from the Walter et al. ^[7]^ study, the estimated sample size was 18 participants utilising the following parameters for two replicate measures: H_0_: p_0_ (minimally acceptable level of reliability) =0.7, H_1_: p_1_=0.9 (maximum expected value of reliability), beta (β) =0.2, alpha (α) =0.05. However, due to multiple tests in the *SCRuM* test battery and the study design involving two repeated measures, over-sampling was done. Of the 59 schoolboy rugby players invited to participate, 41 completed all the tests. Ethical approval was obtained from the Medical Research Council of Zimbabwe (ref: MRCZ/A/2070) and the Human Research Ethics Committee from the University of Cape Town (ref: 016/2016). Institutional permissions were obtained from the Ministry of Primary and Secondary Education and Harare Provincial Educational Office, Zimbabwe. Prior to data collection, written informed consent and assent were obtained from the guardians/parents and the children, respectively.

### Data collection approach

A pragmatic “in-season” approach previously used by Enright et al. ^[11]^ was adopted for the study. Specifically, the study sought to determine the reliability of *SCRuM* tests when test-retest assessments are scheduled during training days without disturbing the classes, training schedules, and competitive match days. This approach was more likely to get approval from the coaching staff, parents, and school authorities given the multitude of tests in the *SCRuM* test battery and the repeated measures. The design required all participants to perform the *SCRuM* test items on two separate occasions at the same time and day.

Two familiarisation sessions were conducted to ensure sufficient exposure of the study participants to the *SCRuM* test items. For the second session, eligible participants completed a brief questionnaire which solicited demographic and rugby-related information. Participant testing commenced during the competitive season. This approach ensured that participants had match physical fitness and were close to peak performance. On any day of testing, participants completed the Physical Activity Readiness Questionnaire (PAR-Q) and were excluded if they reported injuries, illness, or health-related conditions aggravated by exertion. Subsequently, eligible participants completed a standardised warm-up procedure before testing. The order of testing was as indicated in Supplementary File 1. A recovery period of 10 minutes was allowed between tests to minimise fatigue-induced effects. The re-test assessments were conducted after seven days, at the same time for each participant.

Two well-trained research assistants conducted all the *SCRuM* tests, except for skinfolds and game-specific skills. The latter tests were conducted by a purposively-recruited anthropometrist and rugby coaches respectively. Each assistant always assessed the same athlete. Testing occurred on a natural grass pitch for field tests and in a gymnasium for strength/power-based tests with participants who were requested to wear the same clothing each time. The researchers provided similar verbal encouragement to all participants during the test. Test results were deliberately withheld from the participating athletes to avoid influencing re-test performances. Additionally, participants were unaware of the seven-day interval for the re-test assessments and were advised to maintain a normal diet, adequate hydration, and to avoid taking ergogenic aids during the experimental period.

### The *SCRuM* test battery

The *SCRuM* test battery was composed of (i) anthropometric variables (*height, sitting height, body mass, seven skinfold measurements which included biceps, triceps, subscapular, suprailiac, abdomen, thigh, and calf measures*), (ii) physiological characteristics (*speed, agility, upper-and-lower muscular strength and power, prolonged high-intensity intermittent running ability, muscle flexibility and repeated high-intensity exercise performance abilit*y) and (iii) rugby-specific game skills (*tackling proficiency, passing ability, passing-for-accuracy, and running-and-catching ability*). The full description of the SCRuM test battery and methodological procedures followed for the test execution are included as Supplementary File 2. Briefly, linear speed was measured using the 5 m, 10 m, 20 m, and 40 m speed tests. Agility was assessed using the L-run agility test. Upper and lower body muscular strength was assessed using the One Repetition Maximum Bench Press test and One Repetition Maximum Back Squat test, respectively. Two further tests were also included to assess upper and lower body muscular strength: Wall Sit Leg Strength, and 60s-Push Up. Upper-and lower muscular power were assessed using the Vertical Jump and 2 kg Medicine Ball Chest Throw tests. Prolonged high-intensity intermittent running ability was evaluated using the Yo-Yo Intermittent Recovery Level 1 test. Lower back and hamstring muscle flexibility were assessed using the sit-and-reach test. The repeated high-intensity exercise performance ability of the participants was evaluated using the Repeated High Intensity Exercise (RHIE) tests.

The development of the *SCRuM* test battery was based on recommendations from the literature on instrument or test battery development. Briefly, the development process followed a multi-phased approach which involved conducting:

- A narrative literature review to establish what is known about the key requirements of rugby, specifically targeting anthropometric, physical or physiological characteristics, and rugby-specific game skills in the literature
- A qualitative exploratory study to gather the perceptions of rugby coaches on the key attributes or qualities and game skills needed in rugby and should be incorporated in test batteries for TID programs. This part of the approach also sought commonly used test(s) for the identified attributes and skills used in the local context
- A systematic literature review to determine the physical or physiological characteristics and rugby-specific game skills frequently covered in the literature and their corresponding tests. Furthermore, the evaluation of the
- psychometric properties of each identified test per construct was also undertaken.

The above-mentioned processes engendered the first version of the test battery which was subsequently evaluated for face validity, logical validity and practical feasibility. Therefore, this present study aims to evaluate the reliability of the content-validated and practically-feasible version of the *SCRuM* test battery.

### Statistical analysis

Data analysis was carried out using SPSS statistical software version 26. The Shapiro-Wilk test assessed the normality of continuous variables (p<0.05). Descriptive statistics (Mean±SD) described parametric data. Paired samples *t*-test evaluated any systematic bias between trials (p<0.05). Cohen’s *d* effect size (ES) statistic determined the magnitude of the difference between test-retest measurements.^[9]^ The criteria for interpreting the magnitude of the ES were as follows: <0.2 trivial, 0.2–0.6 small, >0.6–1.2 moderate and >1.2 large. Relative reliability was determined by calculating the two-way random intra-class correlation coefficient (ICC 2, 1) for absolute agreement of single measures. However, average ICC measures were reported for the tackling proficiency test because an average of six test trials were recorded to represent participant tackling score (See Supplementary File 2). The 95% confidence interval (CI) was calculated testing if each ICC was equal to zero using the F-ratio. ICC values above 0.7 were considered acceptable for test-retest reliability. ^[10]^ To test for absolute reliability, the standard error of measurement (SEM) was calculated for each test. The SEM provides expected trial to trial measurement error and was computed as a standard deviation of the differences (SD_differences_) between test-retest assessments divided √2. ^[11]^ To facilitate the comparison of test reliability values between studies, the coefficient of variation (CV %) expressed the SEM as a percentage of the grand mean ^[12]^, and an arbitrary CV boundary of <10% was considered acceptable ^[12]^. The smallest detectable change (SDC95%) for each test was calculated by SEM X 1.96 X √2. ^[13]^

## Results

Table 1 shows the demographics and rugby-related information of all participants. The mean age of the participants was 17.5±0.9 years. The median years of experience playing schoolboy rugby for the participants were five years (Interquartile range, IQR four-five years). There was an equal representation of forward (49%) and backline (51%) players in the sample population. Table 2 shows no systematic changes between trials for most *SCRuM* test items. The ICC, SEM, and SDC_95%_ results for *SCRuM* test items are shown in Table 3. Overall, the ICCs for the *SCRuM* test items ranged between 0.49 and 1.0. Evidence of low test-retest reliability was found for the five m speed (ICC=0.52; 95% CI=0.27–0.71), 10 m speed (ICC=0.64; 95% CI=0.42–0.79) and passing-for-accuracy seven m tests (ICC=0.49; 95% CI=0.22–0.69). The SR test exhibited the greatest variability with a CV of 17%. The SDC_95%_ values for all *SCRuM* test items were greater than the SEM values.

## Discussion

The purpose of this present study was to provide contextual evidence on the test-retest reliability of each of the component test items in the newly-assembled *SCRuM* test battery. The establishment of reliability is an extremely important step in test battery development as it provides information on the capacity of test items to differentiate participants or maintain the same relative order of participants in replicate measures under similar conditions. ^[14]^ The ICC is the most commonly reported sample statistic providing evidence of relative reliability in the literature. It thrives on increased variability in the sample population for the measured construct and decreased measurement error. Among 41 U19 schoolboy players, most *SCRuM* test items demonstrated no systematic bias, low CV% values, and high ICCs, suggesting absolute and relative reliability when the assessments are made during the ‘in-season’ phase. These results reflect the careful manner in which *SCRuM* test items were implemented as well as temporal stability in the construct over the interval measured. Overall, the high ICCs could be attributed to the large between-subject variability observed for most test performances. This variability could potentially stem from natural differences in participant abilities, player position heterogeneity, or varied rugby experience.

As expected, good to excellent ICCs were shown for all anthropometric variables. However, 12 of the 14 physiological tests administered to U19 schoolboy rugby players showed good to excellent relative reliability. The tests included the following: 20 m and 40 m speed, modified L-run agility, VJ, SR, 60 s push-ups, 2 kg MBCT, WSLS, RHIE, 1 RM BS, 1 RM BP, and the Yo-Yo IRT L1. Three tests (tackling, passing and catching) of the four rugby-specific game skills assessed had acceptable reliability. These findings suggest that all these tests warrant inclusion in the *SCRuM* test battery for possible profiling of U19 male adolescent rugby players provided there is adequate participant familiarisation and test standardisation. The 5 m and 10 m sprint tests showed low relative reliability among U19s. This questions the appropriateness for the inclusion of these speed tests in the *SCRuM* test battery for the respective age categories given the wide CI. Furthermore, the SEM of each speed test ranged from 0.0 to 0.1 seconds indicating error in consistencies across the speed distances. However, expressed as CV%, the SEM increased with short speed distances and decreased with longer distances. For example, the CV% for the 10 m speed test was 4.5 compared to 1.0 for the 40 m speed test. These findings possibly indicate that the 40 m speed test is more reliable compared to the 10 m speed test among U19 rugby players. Alternatively, the 20 m speed test was more reliable (CV%=1.8) compared to the 10 m speed test but less reliable for the 40 m speed test. These findings of high reliability for longer sprints (above 20 m) among U19s are comparable with previous findings reported elsewhere ^[15]^. Dobbin et al. ^[11]^ reported ICC (CV %) of 0.69 (4.9) for 10 m speed test among 50 U19 academy rugby league players. However, besides differences in sample size and sport, there were methodological differences between the Dobbin et al. ^[11]^ study and our study (i.e. use of timing gates vs an electronic handheld stopwatch; three repeated measures vs two repeated measures). In contrast, Gabbett et al. ^[16]^ reported high ICCs (CV %) for 5 m and 10 m speed tests of 0.84 (3.2) and 0.87 (1.9) respectively among 42 adult rugby league players. This shows that methodological, sport and population differences partly explain differences in the ICC results between studies. Reliability parameters depend on variations in the population sample for the measured construct, and the results have an external validity to populations with similar variations. ^[13]^

Another key but unexpected finding was the low relative reliability for the passing-for-accuracy seven m test. This is explained by the lower variability between participants evidenced by smaller standard deviations in test and re-test scores. No previous study has reported the relative reliability of the passing-for-accuracy seven m test for U19 schoolboy rugby players referencing ICCs values. Pienaar et al. ^[17]^ reported test-retest correlations (r=0.66) and 95% Limits of Agreement (LoA), suggesting moderate reliability among thirty-six 10-year-old schoolboys with varied rugby experience. Nonetheless, the use of *r* has been criticised in contemporary literature since it evaluates the linearity of test scores in repeated measures. ^[18]^ Instead, the ICC is frequently reported for relative reliability. ^[19]^ Nonetheless, the low reliability of the passing-for-accuracy seven m test in the present study could be linked to test novelty. Unlike previous tests which had stationary rugby participants passing a ball to a static object placed seven m away, and judging the accuracy of hitting the target ^[17, 20]^, the present study had a dynamic recipient catching of a pass from a running player. The test also uniquely included a research assistant offering standardised defensive play to the tested player. All this was designed to test passing-for-accuracy as an open skill simulating real game situations. However, given the low reliability, it is possible that the test was relatively easy for U19 rugby players to achieve consistent discriminative performances. To minimise measurement errors, critical test elements, such as the running velocity of the tested and target player and positioning in the passing grid zone for executing the pass, may need careful consideration in future modifications of the test.

All *SCRuM* tests showed acceptable variability (CV<10%), indicating good agreement between test-retest scores, except for the sit-and-reach test among U19 rugby players. The sit-and-reach test showed the greatest variability (CV=17.3%) and paired samples t-test results showed almost statistically significant differences between test-retest assessments (p=0.05). Thus, it is possible that the sit-and-reach test lacked standardisation resulting in the observed mean scores between test-retest assessments or more careful standardisation with the warm-up is required before the test is undertaken. With a mean difference between trials of −0.63, the learning effect could have potentially influenced test-retest results for the sit-and-reach test. This possibly creates a need for an extra familiarisation trial for the sit-and-reach test in future studies or more than two repeated measures.

### Critical assessment of the study

The study utilised a relatively larger sample size than commonly used in similar studies reporting the reliability of anthropometrical and performance tests in rugby. The response and test completion rates were high, eliminating the effect of non-participation bias and missing information on test results. However, the study had some limitations.

- We chose a pragmatic approach involving one age category of participants purposively selected from one school and conducted the study during the competitive rugby season. The residual fatigue from training, especially from previous day and competitive matches, could have affected optimal performance from participants.
- During the test-retest study, no attempts were made to standardise the timing, type and quantity of food/fluid intake.

## Conclusion

Among U19 schoolboy rugby players involved in competitive rugby, good to excellent intraclass correlation coefficients were shown for all anthropometric variables. The *SCRuM* physiological and game skills tests administered which showed good to excellent relative reliability and acceptable absolute reliability included: 20 m speed, 40 m speed, L-run, Vertical Jump, 60 s Push Up, 2 kg Medicine Ball Chest Throw, Wall Sit Leg Strength, Repeated High Intensity Exercise, One Repetition Maximum Back Squat, One Repetition Maximum Bench Press, Yo-Yo Intermittent Recovery Level 1, Tackling proficiency, Passing Ability and Running-and-Catching Ability. All these tests warrant inclusion in the *SCRuM* test battery for possible profiling of U19 schoolboy “elite” rugby players during the ‘in-season’ competitive phase provided there is adequate participant familiarisation and test standardisation.

## Supplementary Information





## Figures and Tables

**Table 1:** Sample demographics and rugby-related information (N=41)

Variable	Elite U19 players
**Age (years)**	
Mean ± SD	17.5 ± 0.9
Range (minimum–maximum)	15.6 – 18.9
**Playing experience years (median (IQR))**	5 (4–5)
**Generic positions**	
Forwards, n (%)	20 (49)
Backs, n (%)	21 (51)
**Specific regular positions**	
Props	7
Flanks	5
Locks	5
Centres	5
Fullbacks	4
Scrumhalf	4
Wingers	4
Fly half	3
Hooker	3
Eighth man	1

SD, standard deviation; IQR, Interquartile range.

**Table 2:** Mean differences in *SCRuM* variables between test-retest results for elite U19s

Variable	Test	Re-test	Mean diff	Std diff	p	ES [95% CI]	Effect
**Anthropometry**							
Body mass (kg)	77.5 ± 9.6	77.6 ± 9.6	−0.1	0.3	0.1	−0.0 [−0.0–0.0]	*Trivial*
Height (m)	1.7 ± 0.1	1.73 ± 0.1	0.0	0.1	0.8	0.0 [0.0–0.0]	*Trivial*
BMI (kgm-2)	25.9 ± 3.3	26.0 ± 3.3	−0.0	0.4	0.5	−0.3 [−0.1–0.1]	*Trivial*
Biceps (mm)	6.7 ± 3.6	6.46 ± 3.4	0.2	0.7	0.0*	0.1 [0.0–0.2]	*Trivial*
Triceps (mm)	9.4 ± 3.0	9.56 ± 2.8	−0.1	1.2	0.5	−0.0 [−0.1–0.2]	*Trivial*
Subscapular (mm)	12.8 ± 2.7	12.9 ± 2.6	−0.1	0.8	0.3	−0.0 [−0.1–0.2]	*Trivial*
Suprailiac (mm)	8.9 ± 3.8	9.02 ± 3.9	−0.1	0.8	0.4	−0.0 [−0.1–0.1]	*Trivial*
Abdomen (mm)	11.4 ± 2.9	11.7 ± 3.2	−0.3	1.4	0.2	−0.1 [−0.3–0.2]	*Trivial*
Thigh (mm)	10.0 ± 2.5	10.0 ± 2.5	−0.0	1.3	0.9	−0.0 [−0.0–0.0]	*Trivial*
Calf (mm)	5.5 ± 1.0	5.54 ± 1.0	−0.1	0.6	0.6	−0.1 [−0.2–0.1]	*Trivial*
Sum of Skinfolds (mm)	64.7 ± 15.6	65.2 ± 15.2	−0.4	2.6	0.3	−0.0 [−0.1–0.1]	*Trivial*
**Physiological tests**							
5m speed (s)	1.1 ± 0.0	1.11 ± 0.0	−0.0	0.0	0.07	−0.3 [−0.8–0.3]	*Small*
10m speed (s)	2.0 ± 0.1	2.03 ± 0.2	−0.0	0.1	0.23	−0.1 [−0.4–0.2]	*Trivial*
20m speed (s)	3.3 ± 0.2	3.22 ± 0.2	0.0	0.1	0.06	0.2 [−0.2–0.5]	*Trivial*
40m speed (s)	5.6 ± 0.3	5.58 ± 0.3	0.0	0.1	0.11	0.1 [−0.1–0.2]	*Trivial*
L-run agility (s)	6.2 ± 0.3	6.20 ± 0.3	0.0	0.2	0.77	0.0 [−0.1–0.1]	*Trivial*
Vertical jump (cm)	47.8 ± 3.8	48.2 ± 3.8	−0.3	1.4	0.15	−0.1 [−0.3–0.1]	*Trivial*
Sit-and-reach (cm)	7.9 ± 5.1	8.51 ± 4.9	−0.6	2.0	0.05	−0.1 [−0.4–0.2]	*Trivial*
2-kg MBCT (m)	9.3 ± 1.3	9.41 ± 1.3	−0.2	0.6	0.05	−0.1 [−0.4–0.1]	*Trivial*
60-s push-up (s)	49.7 ± 10.0	50.7 ± 10.6	−1.0	3.7	0.08	−0.1 [−0.3–0.1]	*Trivial*
Wall Sit Leg Strength (sec)	146.1 ± 9.7	147.9 ± 8.3	−1.9	4.1	0.01*	−0.2 [−0.6–(−0.1)]	*Small*
1-RM Back Squat (kg)	98.4 ± 14.8	98.8 ± 13.7	−0.4	2.9	0.35	−0.0 [−0.1–0.1]	*Trivial*
1-RM Bench Press (kg)	90.5 ± 16.4	90.7 ± 15.7	−0.4	3.4	0.51	−0.0 [−0.0–0.0]	*Trivial*
RHIE (s)	39.3 ± 3.0	39.7 ± 2.7	−0.4	1.8	0.20	−0.1 [−0.4–0.2]	*Trivial*
Yo-Yo IRT (m)	1505.9 ± 75.0	1522.4 ± 87.0	−16.6	60.6	0.09	−0.2 [−0.6–0.3]	*Small*
**Game skills tests**							
Tackling (%)	87.9 ± 8.4	89.5 ± 8.6	−1.6	6.0	0.10	−0.2 [−0.6–0.2]	*Trivial*
Passing Ability (au)	116.2 ± 2.1	116.5 ± 1.5	−0.3	1.4	0.18	−0.2 [−0.5–0.1]	*Trivial*
Pass Accuracy (%)	89.3 ± 7.3	90.7 ± 5.6	−1.5	6.6	0.16	−0.2 [−0.6–0.2]	*Small*
Catching (au)	74.0 ± 1.1	74.2 ± 0.9	−0.2	0.8	0.07	−0.2 [−0.6–0.2]	*Small*

Data are expressed as mean ± SD.

* indicates significant difference (p<0.05).

Mean diff, Mean difference (test score-retest score); Std diff, Standard deviation difference; df, degrees of freedom; p value, 2 tailed probability value; ES, Cohen’s d effect size statistic with the 95% confidence interval; 2-kg MBCT, 2 kg medicine ball chest throw; 1-RM, one repetition maximum; RHIE, repeated high intensity exercise; Yo-Yo IRT, Yo-Yo intermittent recovery test; Tackling (%), Tackling proficiency test; m, metres; kg, kilograms; s, seconds; au, arbitrary units; cm, centimetres; Catching, running and catching ability test.

**Table 3:** Measures of reliability for the *SCRuM* test items among elite U19s

Variable	ICC [95% CI]	SEM [95%CI]	CV (%) [95%CI]	SDC [95%CI]
**Anthropometry**				
Body mass (kg)	1.00 [0.99–1.00]	0.19 [0.09–0.31]	0.24 [0.10–0.35]	0.52 [0.39–0.78]
Height (m)	0.97 [0.95–0.99]	0.01 [0.01–0.03]	0.56 [0.43–0.67]	0.03 [0.03–0.05]
Biceps (mm)	0.98 [0.95–0.99]	0.52 [0.10–1.10]	7.88 [6.60–9.00]	1.44 [1.24–1.67]
Triceps (mm)	0.92 [0.85–0.95]	0.84 [0.20–1.40]	8.84 [6.34–9.10]	2.33 [2.08–2.55]
Subscapular (mm)	0.96 [0.92–0.98]	0.55 [0.42–0.64]	4.29 [3.12–5.13]	1.53 [1.01–1.89]
Suprailiac (mm)	0.98 [0.96–0.99]	0.54 [0.20–1.11]	6.05 [4.20–7.03]	1.51 [1.34–2.16]
Abdomen (mm)	0.89 [0.81–0.94]	0.99 [0.60–1.35]	8.57 [6.12–8.89]	2.74 [2.23–3.17]
Thigh (mm)	0.86 [0.75–0.92]	0.94 [0.30–1.50]	9.43 [7.24–9.89]	2.61 [2.03–2.87]
Calf (mm)	0.81 [0.66–0.89]	0.45 [0.20–0.68]	8.09 [7.01–9.27]	1.24 [1.01–1.45]
Sum of Skinfolds (mm)	0.99 [0.97–0.99]	1.86 [1.04–2.68]	2.86 [1.20–3.45]	5.15 [3.14–6.17]
**Physiological tests**				
5m speed (sec)	**0.52 [0.27–0.71]**	0.02 [0.01–0.02]	1.94 [1.48–2.40]	0.06 [0.04–0.06]
10m speed (sec)	**0.64 [0.42–0.79]**	0.09 [0.06–0.09]	4.50 [3.60–5.40]	0.25 [0.10–0.35]
20m speed (sec)	0.90 [0.81–0.94]	0.06 [0.04–0.07]	1.83 [1.20–2.40]	0.16 [0.08–0.24]
40m speed (sec)	0.97 [0.94–0.98]	0.05 [0.03–0.05]	0.95 [0.35–1.55]	0.15 [0.08–0.21]
L-run agility (sec)	0.90 [0.82–0.95]	0.11 [0.07–0.15]	1.72 [1.10–2.30]	0.30 [0.19–0.37]
Vertical jump (cm)	0.93 [0.88–0.96]	0.97 [0.54–1.14]	2.03 [1.03–2.90]	2.70 [1.90–3.46]
Sit-and-reach (cm)	0.91 [0.84–0.95]	1.42 [1.08–1.57]	17.3 [11.1–19.2]	3.93 [2.89–4.17]
2-kg MBCT (m)	0.89 [0.80–0.94]	0.42 [0.20–0.67]	4.48 [3.40–6.10]	1.16 [0.98–1.27]
60-s push-up (sec)	0.93 [0.88–0.96]	2.59 [1.34–3.07]	5.15 [3.12–7.04]	7.17 [6.07–7.98]
Wall Sit Leg Strength (sec)	0.88 [0.76–0.94]	2.90 [1.87–3.98]	1.98 [0.78–2.94]	8.05 [6.78–9.34]
1-RM Back Squat (kg)	0.98 [0.96–0.99]	2.08 [1.45–3.45]	2.11 [1.34–2.95]	5.77 [3.57–6.17]
1-RM Bench Press (kg)	0.98 [0.96–0.99]	2.40 [2.02–2.67]	2.64 [2.54–2.87]	6.64 [5.67–7.12]
RHIE (sec)	0.79 [0.65–0.89]	1.28 [0.65–1.71]	3.24 [2.10–4.12]	3.55 [3.34–3.98]
Yo-Yo IRT (m)	0.72 [0.53–0.84]	42.88 [34.2–67.4]	2.83 [2.09–3.14]	118.87 [101.1–137.9]
**Game skills tests**				
Tackling (%)	0.86 [0.74–0.93]*	0.84 [0.35–1.40]	4.75 [2.13–5.13]	2.34 [1.67–3.01]
Passing Ability (au)	0.71 [0.52–0.83]	0.98 [0.30–1.50]	0.84 [0.45–1.23]	2.71 [2.01–3.14]
Passing Accuracy (%)	**0.49 [0.22–0.69]**	4.66 [3.40–5.70]	5.17 [3.76–7.12]	12.91 [9.03–14.78]
Catching (au)	0.70 [0.37–0.81]	0.54 [0.10–1.00]	0.72 [0.24–1.03]	1.49 [1.04–2.17]

Bold indicates low ICC values. The units for the SEM are the same as the variable. ICC, intraclass correlation coefficient; 95% CI,95% confidence interval; SEM, standard error of measurement; CV, coefficient of variation; SDC, smallest detectable change;

* ICC value expresses absolute agreement for average measures;

BMI, Body mass index, 1-RM, one repetition maximum; Yo-Yo IRT, Yo-Yo intermittent recovery test; RHIE, repeated high intensity exercise; au, arbitrary units; catching, running and catching ability skills test.

## Data Availability

All relevant data are within the paper and its supporting information and supplementary files.
