# Supplementary material for: Absolute and relative reliability of SCRuM test battery components assembled for schoolboy rugby players playing competitive rugby in low-resource settings: A pragmatic in-season test-retest approach
Source: S Afr J Sports Med. 2021 Feb 15;33(1):v33i1a12220. doi: 10.17159/2078-516X/2021/v33i1a12220 (PMC10883676; doi:10.17159/2078-516X/2021/v33i1a12220)
Supplement: Supplementary file 1 [file 2078-516X-33-v33i1a12220-s001.pdf]

Absolute and relative reliability of *SCRuM* test battery components assembled for schoolboy rugby players playing competitive rugby in low-resource settings: A pragmatic in-season test-retest approach

Supplementary file 1: Order of the SCRuM tests performed during test-retest reliability study for elite Under 19 schoolboy rugby players

Group	Week	Monday	Tuesday	Wednesday	Thursday	Friday	Saturday	Sunday
Elite U19 (n=41)	Week 1	Body mass<br>Height<br>7 Skin folds<br>Sitting height	Yo-Yo<br>2kg MBCT	1RM BP<br>WSLG<br>1RM BS<br>VJ	Speed<br>SR<br>L-run	RHIE<br>Push Up	Match	Rest
	Week 2	Body mass<br>Height<br>7 Skin folds<br>Sitting height	Yo-Yo<br>2kg MBCT	1RM BP<br>WSLG<br>1RM BS<br>VJ	Speed<br>SR<br>L-run	RHIE<br>Push Up	Match	Rest
	Week 3	Tackling	Passing		Catching		Match	Rest
	Week 4	Tackling	Passing		Catching		Match	Rest

Yo-Yo, Yo-Yo Intermittent Recovery Level 1 Test; 2kg MBCT, 2kg medicine ball chest throw tests; 1RM BP, One repetition maximum bench press test; 1RM BS, One repetition maximum back squat test; WSLG, Wall Sit Leg Strength test; VJ, Vertical Jump test; SR, Sit-and-Reach test; Push Up, 60s push up test; RHIE, Repeated High Intensity Exercise Performance Ability test; Match, Represents competitive match; 7 skin folds, biceps, triceps, subscapular, suprailiac, abdomen, thigh, and calf measures.; Tackling, Tackling proficiency test; Passing, Passing ability and passing for accuracy for 7m test; Catching, Running and Catching Ability test.
