# Supplementary material for: Absolute and relative reliability of SCRuM test battery components assembled for schoolboy rugby players playing competitive rugby in low-resource settings: A pragmatic in-season test-retest approach
Source: S Afr J Sports Med. 2021 Feb 15;33(1):v33i1a12220. doi: 10.17159/2078-516X/2021/v33i1a12220 (PMC10883676; doi:10.17159/2078-516X/2021/v33i1a12220)
Supplement: Supplementary file 2 [file 2078-516X-33-v33i1a12220-s002.pdf]

1. Height/Stature (cm)

Height was measured using a portable stadiometer (Seca Model 213, Hamburg Germany). Participants wore shorts and rugby training shirts. Participants stood on the base of the stadiometer with the feet placed together, arms hanging by the sides and looking directly forward. The heels, buttocks, upper back, and head in touch with stadiometer. The testing procedure involved lowering the stadiometer to the top of the participant’s head and noting the height of the participant to the nearest 0.1cm. Giving 30 seconds “rest”, the participant was measured again using the same procedure for the second measurement. The mean of the two measurements was recorded.

2. Body mass (kg)

Body mass was measured to the nearest 0.1kg using calibrated Seca 813 high capacity digital flat scale. The participant would stand on the scale with no movement and arms hanging by the sides of the body wearing light clothing (shorts and rugby training shirt) with no shoes or socks. After the first reading, 30 seconds of “rest” were allowed and the participant was measured again for the second reading. The mean of the two measures was recorded as participant body mass.

3. Skinfold measurements (mm)

The sum of seven site skinfolds (biceps, triceps, subscapular, suprailiac, abdominal, thigh and calf) was determined using calibrated Lange skinfold calipers to indicate body composition. The same researcher performed all the skinfold measurements. Participants were measured on the right side of the body. Participants would stand in the anatomical position with shirt removed. The research assistant would identify a landmark pint using a tape measure and mark with a fine point felt tip pen order of the testing followed was: biceps, triceps, subscapular, suprailiac, abdomen, thigh and calf. The specific positioning for taking the skinfold measurement of each site was in accordance with procedures previously described in literature. However, the general procedure involved gently and firmly “pinching” the skin and the subcutaneous fat between the thumb, forefinger and middle finger. The researcher would then open the skinfold caliper and measure the skinfold approximately 1cm

below the finger and 1cm deep into the skinfold. The researcher would only release the caliper from the skinfold after obtaining the skinfold measurement. Two measurements per site were measured and had to agree within 1 millimeter otherwise subsequent measurements had to be taken until all values are within 1 millimeter.

4. Sitting height (cm)

The participants sat on a chair with hands resting on thighs. Minimal clothing was allowed (shorts and rugby training shirt). The research assistant would push the stadiometer down to vertex of head then record measurement to nearest 0.1 cm. The participant would stand up and re-measured again after 30 seconds. Sitting height represents stature height minus chair height. Age at peak height velocity (APHV) was deduced from this equation given by Mirwald et al: -9.326 + (0.0002708 x [leg length x sitting height]) – (0.001663 x [age x leg length]) + (0.007216 x [age x sitting height]) + 0.02292 x [weight/height]. The prediction equations to determine APHV seem to be a valid method of assessing the biological maturity status of youths.

5. Speed tests (5m, 10m, 20m, 40m)

The course was set-up using the measuring tape (measuring wheel). Participants wore shorts and t-shirts, and were allowed to run in their rugby shoes. A pre-test warm procedure was conducted first for 2 minutes, including shoulder-arm stretches, trunk rotation stretches with hands on the waist, bilateral quadriceps femoris stretches in standing, toes-touch stretching and jogging. The four distances were assessed independently and each participant was assessed at one point in time to completion of the tests. The starting procedure would be demonstrated by the research assistant and the participants allowed pre-test practice trials until the player understood the procedure.

The participants ran the 5m distance first, followed by the 10m, 20m and finished the sprint test on the 40m speed test (illustration 1). Participants would start from a stationary position, with one foot in front of the other. The front foot started precisely 50cm behind the starting line. The players would set off in their own time and run maximally through the specified distance. The criteria used to determine

the completion of the sprinting segment was the first body part to cross the finish line. Two test trials were run for each distance and the better of the two was recorded for each distance. The first research assistant at the starting line was judging if the start was valid and the second research assistant recorded the time taken. All the test trials with a false start were repeated. Markers were left on the pitch to ensure for identical placement of cones between sessions. All the times were recorded with a hand-held digital stopwatch. The JUNSD digital stopwatch (model JS-306) was used.

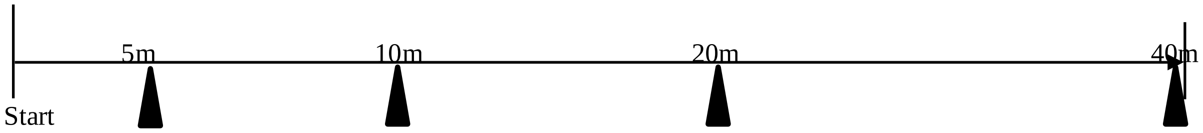

Illustration 1: Schematic representation of speed tests

6. L-run agility test (sec)

This was conducted as previously described in literature with minor modification. Three cones were placed 5m apart from each other to represent an ‘L’ shape. A pre-test warm procedure was conducted first for 2 minutes, including shoulder-arm stretches, trunk rotation stretches with hands on the waist, bilateral quadriceps femoris stretches in standing, toes-touch stretching and jogging. The starting procedure would be demonstrated by the research assistant and the participants allowed pre-test practice trials until the player understood the procedure. Players would start at a line 1m from the first cone lying in a prone position (chest down) mimicking a rugby player coming from a physical collision, ruck or maul. At the first cone, a rugby ball was placed and participants were instructed to get up on “GO” command and pick up the rugby ball and run as quickly as possible along the 5m, turn left, run forward in between the cones, turn 180 degrees, run straight to finish and score a “try” or drop the ball at the 1m line. Hand-held stopwatches were used to record the time from the start to the finsh when the ball was dropped on the 1m line. Two trials were done and the best count was recorded. Additional trials were given when the participants failed to grab the rugby ball at the first cone, failed to execute the zigzag between the cones or droping the ball at the 1m line or the research assistant adjudicates that the prone lying at the start of the Lrun agility test was not perfect.

7. **Vertical jump (VJ) test (cm)**

Participant wore shorts and t-shirts and performed the test barefooted. A warm up procedure as explained for the sprint test was conducted first. The research assistant would demonstrate the testing procedure and participants allowed to practice with a maximum of 3 practice trials. Players stood with fleet flat, shoulder width, on the ground, with the wall sideward to their dominant side. Participants extended arm and hand and mark the highest possible point with the chalk as mentioned in Gabbett 2000 study. The player went to a crouched position. They were encouraged to crouch deeply, close to 90 degrees of active knee flexion, while keeping the feet flat on the ground. They were supposed to hold a new full-length chalk between the tips of the index and middle fingers every time and spring upward as high as possible and mark the highest point possible on the wall. The research assistant would draw a small line with a ruler on top of the dot made by the chalk representing the maximum height reached. 30 seconds of rest were provided in between the 2 trials. The best count was recorded in cm using a tape measure. If the player fell down during the procedure, the trial was re-done and if the crouching was too shallow the trial was stopped and re-done.

8. **Sit-and-Reach (SR) test (cm)**

Participants sat on the ground, knees extended, and soles of the feet contacting the rigid sit-and-reach box. Each subject would be asked to stretch as far forward as possible with stacked hands and to hold that position for one second. They were encouraged to go as far along as they can with the hands together and keep knees straight throughout (illustration 2). Each player was allowed 3 maximum practice attempts before test trials. During the test trials, two attempts were made and the best score recorded. A positive score indicated that the tip of the participants’ fingers reached past the zero line indicated on the sit-and-reach test box while a negative score indicated that the tip of the fingers could not reach the zero reference line. During execution of the test, knees were not allowed to bend otherwise the test was restarted.

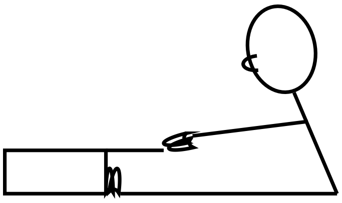

Illustration 2: Schematic figure for the sit and reach test

9. 60 seconds push-up test

Players began in prone, with hands on the floor, thumbs shoulder width apart and elbows fully extended (illustration 3). Players were instructed to descend to the tester fist placed on the ground below the players' sternum and then ascend until the elbows are straight. Push-up action was to be continuous with a single rest of no more than 2 seconds permitted between repetitions. Athletes were required to perform the maximum number of push-ups as fast as possible in 60 seconds. If the athlete fails to complete the full 60 seconds due to fatigue, this failure was recorded together with the number full repetitions recorded and the time of drop-out.

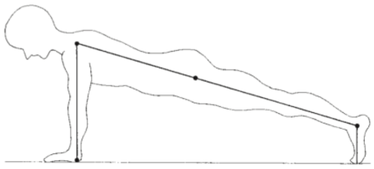

Illustration 3: Schematic for the 60s push up test

10. 2kg medicine ball chest throw (2kg MBCT)

This test was conducted as described in literature to measure upper body power. Players threw a 2kg medicine ball (dimensions=21.5cm) horizontally as far as possible while seated with the back, and legs straight. Distance was measured using the tape measure to the nearest 0.1m from the sitting line to where the ball initially landed with the higher of two trials measured. The two trials were separated by two minutes of recovery. Additional trials were allowed when the knees bent, and if the ball landed outside the circumscribed measuring area.

11. Wall sit leg strength test (WSLS) (sec)

The WSLS assessed static strength of the lower extremities. Participants stood comfortably with feet approximately shoulder width apart, with back against a smooth vertical wall. Participants would be instructed to slide the back down maintaining contact with the wall to assume a position with both knees and hip of 90° angle. However, the timing started when one foot was lifted off the ground and was stopped when the subject could not maintain that position and the foot is returned to the ground. The non-dominant leg was lifted to measure the leg strength of the dominant side.

12. Repeated high-intensity exercise test (RHIE) (sec)

The RHIE test was devised in two parts as provided by Austin et al [33]. There was one for the backline players and the other test for forward players.

Back test

For backline players, each player started with the toes behind a marker. They then completed three 20-m sprints before decelerating and jogging to the start (illustration 4). The sprints were performed on a 20-second turnaround, with approximately 16–17 second active recovery. The players were instructed to sprint with maximal effort. After completing 3 sprints (and after a 60-second recovery), the player moved to the side of the running lane and completed 2 tackles. They sprinted 10m to the tackle a bag (junior bag 117cm by 38cm, 15kg) held by a research assistant, driving it for 2 m. They then ran backward to the start and 20 seconds later completed a second tackle. A 20-second recovery followed the tackle shuttle; the player then repeated the 3 by 20-m sprint protocol and tackles drill.

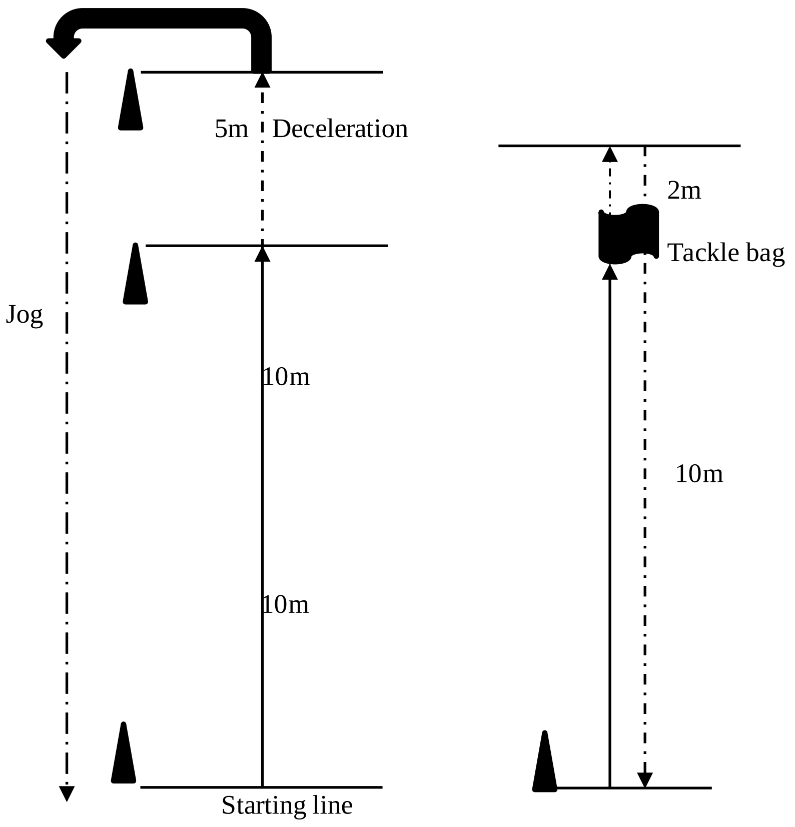

Illustration 4: Schematic of backline players RHIE test

Forward test

Each player first completed 3 by 20-m sprints before decelerating and jogging to the start (illustration 5). Each participant was instructed to sprint to the best of their abilities. Each sprint was performed on a 20-second cycle, leaving 16- to 17-second recovery between sprint efforts. After the third sprint in each set and after a 60-second rest, the player moved to complete a "scrum sled shuttle". This involved pushing a weighted (50 kg) one man scrum sled for 5 m in 1 direction and 5 m back to the start. The player completed this 4 times, with a 10-second rest between bouts. On completion of the scrum sled shuttle, the player was given 20 seconds to return to the sprint lane. Participants would then repeat the sprint shuttles (3 by 20 m) before moving to the tackle drill. After a 60-second rest, he sprinted 10 m and tackled a tackle bag driving it at least 2m. On completion of the tackle, players ran backward to the start line and repeated the tackle drill on 4 occasions; 20 seconds separated the start of each sprint to tackle. A final set of 3 sprints then concluded the test. Times were recorded for each sprint repetition (total of 9).

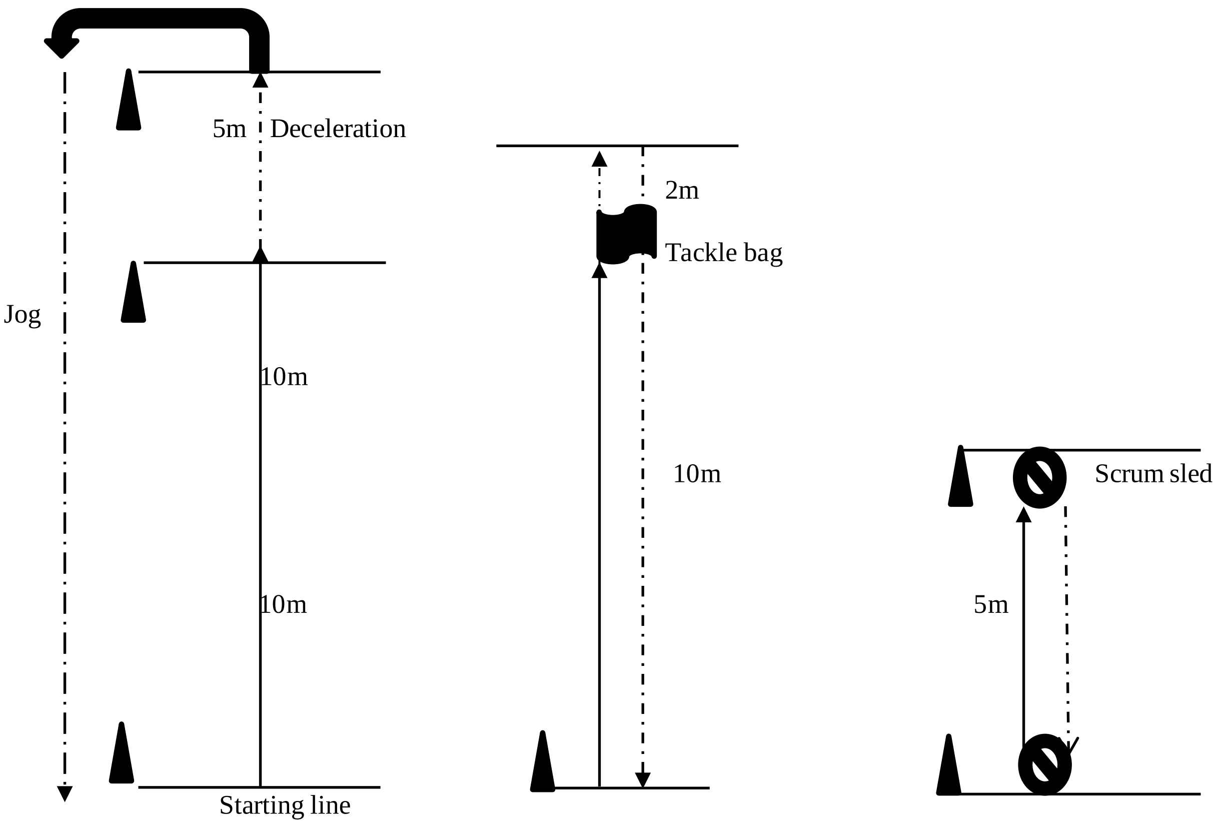

Illustration 5: Schematic of forward players RHIE test

Total RHIE performance sprint time was taken as the sum of all 3 sets of sprints (nine 20-m sprints in total). Decrement in sprint performance was calculated as the difference in time taken (seconds) to complete the third set of sprints (sprints 7–9) compared with the total time taken to complete the first set of 3 sprints (sprints 1–3).

13. Yo-Yo intermittent recovery test level 1 (m)

The Yo-Yo IRT L1 was conducted as previously described in literature. Players were required to run back and forth along a 20-m track, keeping in time with a series of signals on a compact disk. The frequency of the audible signals (and hence running speed) was progressively increased until subjects reach volitional exhaustion. The course was set up on a dry (grass) surface. Participant wore short and rugby t-shirts, and studded shoes for playing rugby. Participants did a proper warm-up for 2 minutes, including shoulder-arm stretches, trunk rotation stretches with hands on the waist, bilateral quadriceps femoris stretches in standing, toes-touch stretching and jogging. Demonstrations were performed and participants were also allowed to try the procedure till they understood test performance.

Starting from a stationary position, participants would run back and forth along the 20m track while keeping in time with the audio signals played from the compact disk. The players started after the beep and runs to opposite line. The players turned when the next beep goes. There were 2 research assistants at both lines to check if the player touches the line with 1 foot and if they keep in time with the audible signals. They also make sure the players don't start running back before the beep the sound. After about 1 minute, a sound indicated an increase in speed, and the beeps got closer together with time. If the line was reached before the beep, the subjects waited until the sound beeps before continuing. If the line was not reached before the beep, the participant was given a warning and must continue to run to the line, then turn and try to catch up with the pace within two more beeps. The test was stopped for that player who failed to reach the line (within 2 meters) for 2 consecutive ends after a warning. The research assistants were checking for this. We recorded the number of complete laps run and shuttles. This was used to calculate the total Yo-Yo distance in meters.

14. One repetition maximum back squat (kg)

All the participants had to be experienced with the squat protocol, having performed it for a minimum of two years before our assessment. Each participant performed two warm-up sets using a resistance that was approximately 40-60% of their estimated 1RM. The participants were required to lower the barbell to a depth equivalent to at least 90 of knee flexion as visually determined by the researcher for the attempt to be considered successful and players had to return to the standing position to record 1RM score. The greater trochanter of the femur had to be aligned with the patella and on ascension the knee and hip had to be in full extension. If the set was successfully completed then weight was added and if not weight was reduced and another set attempted. A 3-5 minutes rest was provided between each set. This process of adding and removing was continued until a 1RM was achieved. The players' 1RM scores were divided by the body mass to provide a strength score relative to body mass.

15. One repetition maximum bench press (kg)

All the participants had to be experienced with the press protocol, having performed it for a minimum of two years before our assessment. Athletes lower the barbell to touch the chest and push the barbell

until the elbows were locked out. Participants used a self-selected hand position and were required to lower the bar at approximately 90 degree angle at the elbows and then pressed the bar in a vertical position so that the arms are fully extended. Participants need not to bounce the barbell off their chest or lose contact between the bench and their hips or the floor and their feet.

16. Tackling proficiency test

This test was modified from previously used tests in literature. A simulated rugby-specific match 2-on-1 scenario was simulated within a 10 × 10 m grid for the tackling test (illustration 6). A local rugby coach with experience served as an expert rater (obs 1) for the skill rating of the participants. The players performed a warm-up led by the lead author consisting of jogging and upper and lower body dynamic stretching. Three players were used at one given time. The two attacking players (ball carriers) were former U19 rugby players recruited as research assistants for this test. They were instructed to advance from one side of the grid to the other and complete one pass each before being tackled by the defending player (test player). The test players were oblivious of the number of passes to be completed between the attacking players to mimic a real rugby scenario which is underlined by unpredictability. The attacking players were set at 4m apart from each other and 5m away from the tackler at the beginning.

The attacking players were allowed to make passes between them within the 3m while advancing forward. A line at 3m indicated where no more passes between the two attacking players were allowed. The test player was initially stationed 5m away from straight line marking the starting line of the attacking players. The procedure was for the test player to tackle the player with the ball. If the attacking player runs so good that the test player does not get him, the test was repeated. Demonstrations were given to enhance players understanding of the test and to provide them with the reference for the required match-like intensity. After one cycle of this protocol the players waited for a brief recovery period (1minute) on their feet at the opposite end of the grid before repeating the drill. Six test trials were conducted to allow the observer to observe the tackling skills under fatigue as well.

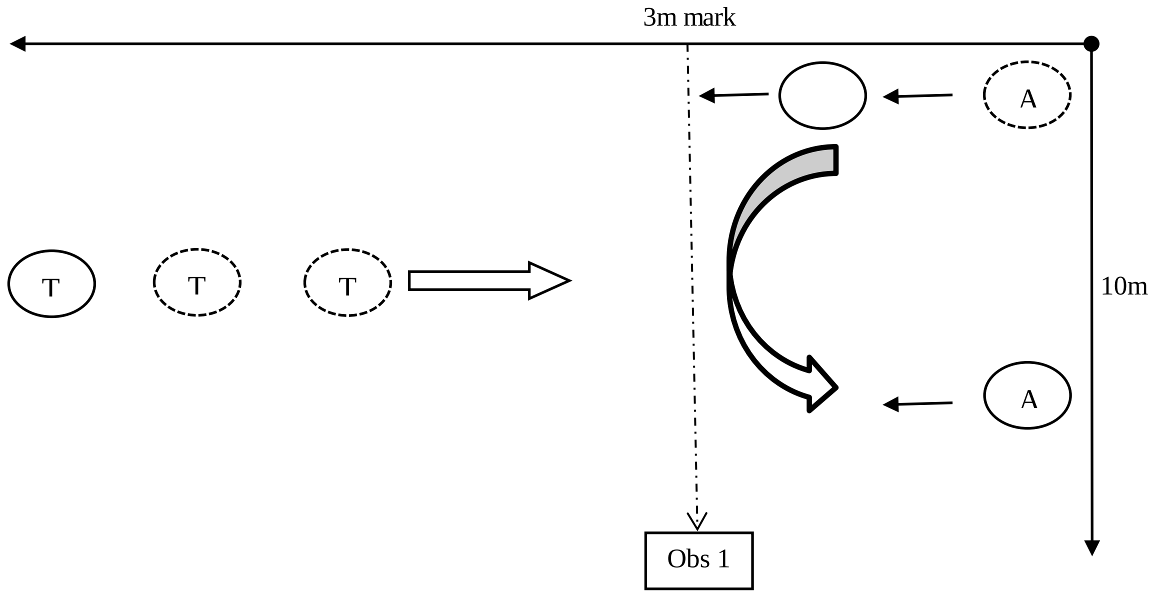

Key

T= Tackling Player Being Assessed; A=Passing and Receiving Player; 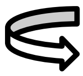 =Ball passing direction

Illustration 6: Tackling protocol

The observer assessed and scored each of those six trials based on technical checklist. The observer rated each player in real time on their overall proficiency in each skill using a Likert scale (0-not achieved completely; 1-partly achieved; 2-completely achieved relevant criteria). The coaches were given the assessment criteria two weeks prior to testing and it was discussed with them in detail. They were given the explicit instruction to refer to the criteria during the testing procedures. The technical criteria were as follows:

1. Contacting the target in the centre of gravity/low body position
2. Contacting the target with at least one shoulder
3. Head to one side of the body
4. Arms should readiness for tackling
5. Body position square/aligned
6. Arms completely wrapped around the target on contact when tackling
7. Leg drive on contact/ drive with the legs
8. Centre of gravity forward on base of support
9. Maintain grip until the attacking player is on ground
10. Turtle the player/ hold the player immobile on the ground/defensive shape

Example of the tackling proficiency scoring system

Code	Test assessment											Retest assessment										
U19PE1	Trial #	1	2	3	4	5	6	7	8	9	10	Sum	1	2	3	4	5	6	7	8	9	10	Sum
	**1**	1	2	1	2	2	2	1	1	2	1	**15**	2	1	2	2	1	1	1	2	2	1	**16**
	**2**	1	2	2	2	2	1	1	2	2	1	**17**	2	1	2	2	1	2	2	1	2	1	**17**
	**3**	2	2	2	2	2	2	2	2	2	2	**20**	2	2	2	2	2	2	2	2	2	2	**20**
	**4**	1	2	2	2	2	1	1	2	1	2	**16**	2	2	2	2	2	2	2	2	2	2	**20**
	**5**	1	2	2	2	2	2	2	1	1	2	**17**	2	2	2	2	2	2	2	2	2	2	**20**
	**6**	2	2	2	2	1	2	2	2	2	2	**18**	2	2	2	2	2	2	2	2	2	2	**20**
	**Av.**											**17**											**19**

As shown above, an average of the tackling proficiency arbitrary scores from the six test trials was calculated to represent the test player test score for tackling. The tackling proficiency arbitrary scores were then expressed as percentage of the total possible scores.

17. Passing-for-accuracy over 7m test and passing ability skill test

This test was used for combined assessment of passing skill ability and accuracy in passing for over a 7m distance modified from previous studies. Only one player was tested at any given time. Participants commenced chest down, flat on the ground, and knees extended behind the starting line (illustration 7). They stood up on the word “go” from a research assistant stationed at the starting line and grab a rugby ball placed on the touch line and sprint in a zig-zag way on a 10m course set out using cones. The participants were instructed to run as quickly as possible. Upon entering the passing grid zone (measuring 3m by 3m) they were supposed to release the rugby ball and prepare to receive a pass from one of the expert coaches (E1) acting as a research assistant for the study. There were instructed to catch the ball and pass immediately at a moving target (R) placed at a 7m distance with a defensive player approaching to offer a hindrance (A). The pass had to be made from the centre of the passing grid zone. Another rugby expert (E2) rated the pass made by the first expert (E1) and if the pass was deemed bad, the test was repeated. The target (R) would have started at the starting line and moved slowly with the pace of the tested player in anticipation of receiving the ball. The target player used was former U19 elite rugby player acting as a research assistant for the research team.

After each pass, the subject ran back to the starting line and repeats the test without starting in prone this time. They would start each 10m sprint at a 20sec cycle. This happened five times making it one set of passing ability assessment. The second set started 60seconds after the completion of the first set and would begin with the participant in prone again, and chest on the ground and repeating everything else alluded above. After completing the second set of five runs, the participant rested again for 60seconds before embarking on the last final set. Overall, the total passes made were 15 executed in three sets of 5. Participants decided on which side they preferred to pass depending on their hand dominance. The number of accurate passes made (passes caught) to the receiver (R) was determined by the lead author observing and expressed as percentage of the total passes made to give the passing accuracy (%) score for the participant. In addition, an expert rugby coach (E2) judged passing ability looking at the eight elements giving a passing ability score for each pass made looking at the technical elements utilised in the pass. The scoring was based on a dichotomous response scale: 0-not achieved; 1-achieved. So each participant was assessed 15 times (3 sets of 5) and a score was recorded for each technical element. All the scores were then added for each test trial to give a total passing skill score reflecting passing ability score in arbitrary units. The technical criteria assessed:

1. Pendulum action
2. Looking where pass is to be made
3. Single movement
4. Straight follow through of passing hand
5. Appropriate ball speed
6. Pass in front of the receiver
7. Receiver catches the ball
8. Receiver maintain stride/minimal breaking of the receiver pace to receive the ball

The detailed version of the technical criteria has been further summarised below

Technical criteria	Summarised version	Detailed explanation
1	pendulum action	Swing ball through in single motion in an 'up and down plane' (not across body)
2	Look where passing	look where passing without breaking the natural pace, postural and kinematic adjustments allowing the body to pass left or right
3	Single movement	Total movement should be achieved in one motion while running straight line
4	Straight follow through of passing hand	Straight follow through of passing hand
5	Ball speed	Appropriate ball speed
6	Pass in front of the receiver	Pass in front of the receiver
7	Receiver catches the ball	The point of a pass is for the receiving player to catch it. Accuracy judged on complete passes received
8	Receiver maintain stride	Minimal breaking of the receiver pace to receive the ball, and should catch the ball within his catching grid zone without accelerating fast to keep up with a fast pass.

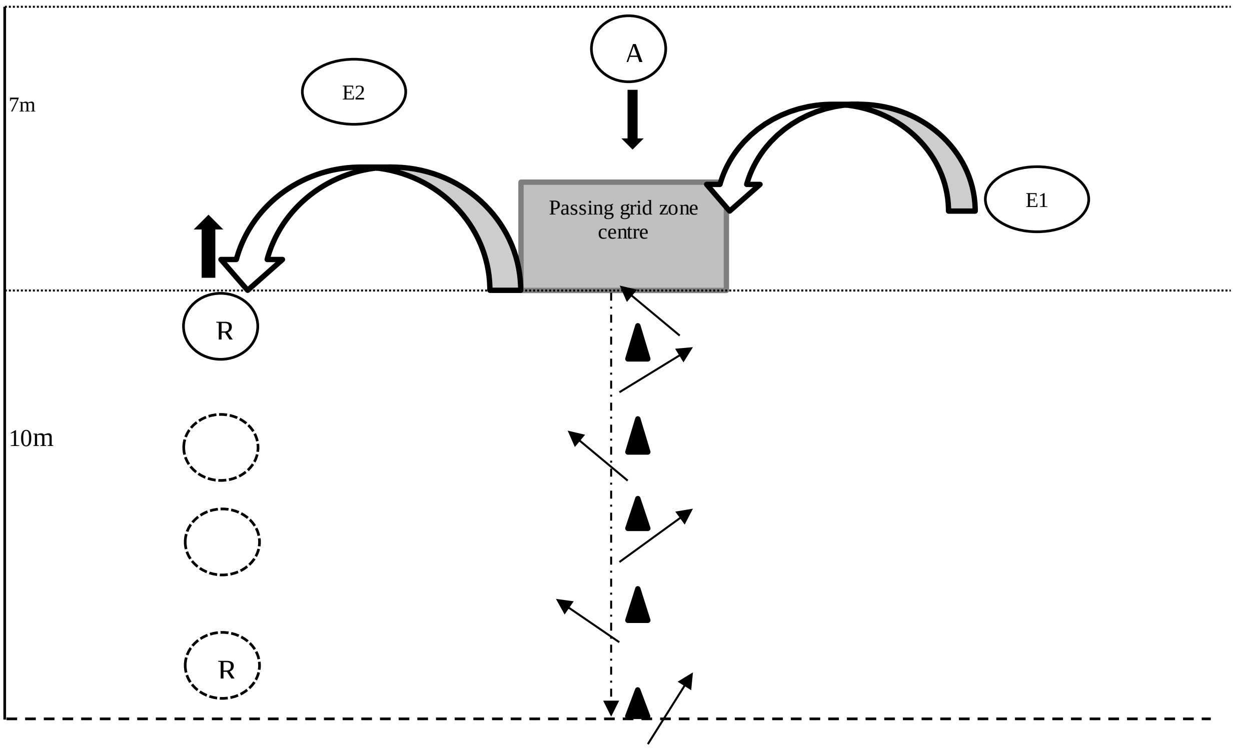

R- Receiver; A-Attacking player; E1- Coach Expert 1(Passer); E2-Coach Expert 2 (Rater)

Illustration 7: Passing protocol for passing ability and accuracy assessment

18. Running-and-catching ability test

The protocol was more or less similar to the passing protocol and was modified from descriptions of Pienaar et al [38] and Gabbett et al [44] and was mainly designed for the assessment of catching ability skills test based on expert rating. Briefly, participants ran in a zigzag fashion for 10m holding the rugby ball. Starting position of prone was similar as for the passing procedure (illustration 8). Upon entering the catching grid zone (measured 3m by 3m) the ball is passed immediately from 7m and the test player should show ability to catch the ball. The test was performed on a different day from the passing protocol to allow for independent assessment of the skills accurately.

Two player assistants providing defensive play were utilised in the protocol. Their duty was to provide cognitive recognition of impending attack to the test player for the passed ball to simulate match rugby situations. Their hidden aim was to compete for the ball as well with the tested player. The two assistants were placed equidistant from participant catching grid. There were also two coach experts acting as research assistants, one rated the technical ability of catching as a skill and the other one was helping with the decision on the quality of the pass made to the test participant. If the pass was deemed not suitable, looking at some of the key things highlighted under passing ability, then that pass will not be rated for catching. Otherwise a re-throw was done. If the pass was deemed acceptable and the test participant misses it was recorded as a missed catch and awarded corresponding scores.

There was a technical criteria used for assessment of running-and-catching ability. Each of the five criteria was assessed based on a Likert scale from 0 (failed completely to perform the activity), 1 (completely achieved). Participants were assessed 15 times (3 sets of 5) just like in the passing protocol. The idea is to see how the participants would fare before and after fatigue has set in. The total score per test trial was 5 aggregating to 75 after completion of 15 tests trial. The technical criteria looked at the following elements:

1. Eyes on the ball/Focus on passer/ Body receptive to the pass
2. Hands up/elbows bent/
3. Fingers spread/palms out and thumbs up
4. Take the pass early/meet the ball early

5. Catch the pass/Hold the "body" of the ball and all this with minimal breaking of the natural or starting stride of the player

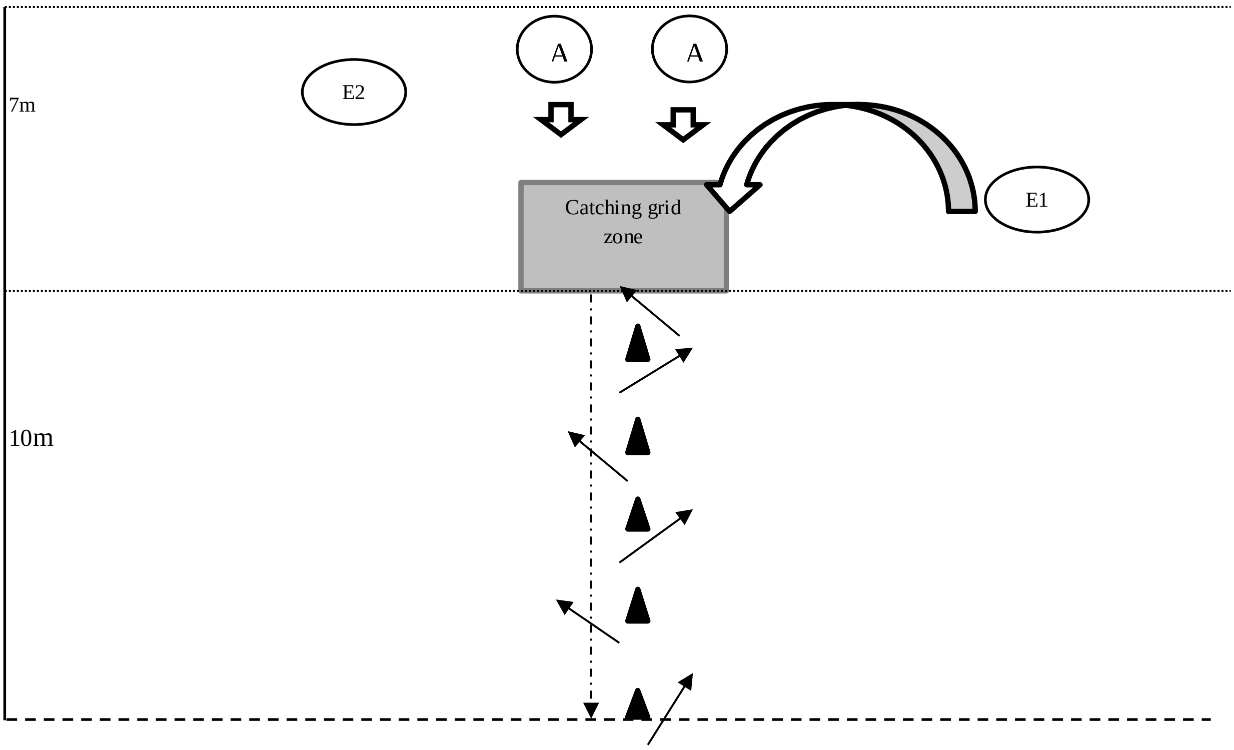

Key: A-Attacking players; E1- Coach Expert 1(Passer); E2-Coach Expert 2 (Rater)

Illustration 8: Running-and-catching protocol for assessment of catching ability skills
